# High-glutathione producing yeasts obtained by genetic improvement strategies: a focus on adaptive evolution approaches for novel wine strains

**DOI:** 10.3934/microbiol.2017.2.155

**Published:** 2017-03-23

**Authors:** Luciana De Vero, Tommaso Bonciani, Alexandra Verspohl, Francesco Mezzetti, Paolo Giudici

**Affiliations:** Unimore Microbial Culture Collection (UMCC), Department of Life Sciences, University of Modena and Reggio Emilia, Italy

**Keywords:** glutathione, yeast, *Saccharomyces cerevisiae*, evolutionary engineering, genetic improvement, winemaking

## Abstract

Glutathione (GSH) is the most abundant non-protein thiol in living organisms. Due to its important antioxidant role, it is widely used in medicine, as a food additive, and in the cosmetic industry. Recently, GSH has received growing attention in winemaking because of its ability to control oxidative spoilage damage and to protect various aromatic compounds. Indeed, GSH concentration in wine is highly variable and several factors are involved in its regulation, ranging from grape must to yeast fermentation activity. This short review aims at highlighting the common genetic strategies, useful for obtaining wine yeasts with enhanced GSH production, paying particular attention to the adaptive evolution approaches. Moreover, other strategies, such as random mutagenesis, metabolic engineering and hybridization have been briefly reviewed with a stress on both their strengths and weaknesses in terms of actual feasibility and acceptance by wine consumers.

## 1. Introduction

In winemaking, as generally in fermented food, the selection of appropriate starter cultures is the key for a successful process. In particular, the use of the appropriate microorganism with desired fermentative and metabolic traits improves the overall fermentation process and leads to a wine of high quality. The most used yeasts applied in winemaking are the species belonging to the *Saccharomyces genus*, such as *S. cerevisiae* and *S. uvarum.* The selection and genetic improvement of wine strains started a long time ago, but it is still in progress, since yeasts with new traits are required on the market [1]. Improvement of yeast strains can be achieved in several ways by exploiting one method over the other according to the complexity of the targeted character and the knowledge of molecular and regulatory interactions, which lie behind a specific desired trait [2].

Generally, the basic traits for good wine strains are fitness and predictable behavior, vigorous fermentation with a short lag-phase as well as tolerance to ethanol and SO_2_. Furthermore, the avoidance of yeast-borne off-flavors is particularly important, together with other traits relevant for specific wines [3],[4]. In addition to the previous traits, there has recently been an ever-growing interest in glutathione (GSH)-producing yeasts. GSH is a tripeptide formed by L-cysteine, L-glutamate and glycine, which is found in both eukaryotic and prokaryotic cells in concentrations varying from 0.2 mM to 10 mM [5]. It is characterized by a very low redox potential (E_0_ = –240 mV) and by a peculiar γ-glutamyl bond, involving the carboxylic function of the glutamate side chain and the amine group of cysteine, that is not recognized by peptidases, thus allowing this compound to reach high cytoplasmic concentrations [6]. Moreover, another important characteristic of GSH is that its reduced state is actively sustained by a NADPH-dependent glutathione reductase [7].

## 2. Significance of Glutathione in Oenology

Glutathione is a natural antioxidant contained in grapes as well as in yeasts, where it plays similarly important physiological and biochemical roles. In particular, GSH is involved in redox control, detoxification and sulfur metabolism. Furthermore, GSH can exert various activities in must and wine, ranging from the preservation of important varietal aroma compounds to the limitation of atypical ageing off-flavors in wines [8].

### 2.1. Glutathione in winemaking

The role of GSH in winemaking is strictly connected to its redox properties. In fact, GSH is involved in the prevention of the browning reactions, which can occur in must as a result of enzymatic or non-enzymatic reactions, both involving phenols [9]. The antioxidant character of GSH exerts a protective role in wine: it stabilizes the color of the product by inhibiting the polymerization of phenolic compounds [10],[11]. In addition, GSH can prevent the formation of sotolon and aminoacetophenone, atypical aging characters in wine [12], and can preserve the aroma compounds by avoiding the loss of volatile thiols, esters and terpenes [13],[14]. Thus, the usage of GSH in winemaking can partly replace sulfur dioxide as antioxidant, imparting beneficial effects in terms of health for the consumer.

GSH is naturally contained in grape must and wine; its amount varies a lot among the different cultivars and the final content depends on many factors involved in the fermentation process. The concentration values of GSH range from non-detectable to 100 mg L^−1^ in must and from non-detectable to 70 mg L^−1^ in wine; however, literature shows that these values generally tend to be as low as a few mg per liter [8]. Dubourdieu and Lavigne [12] observed that the GSH content in the matrix decreases with the beginning of alcoholic fermentation and then increases again because of cell lysis and *ex-novo* synthesis by yeasts. The yeast itself is capable of either secreting GSH in the extracellular environment or even assimilating it from the must, depending on many factors including process conditions and even the metabolic content of the initial raw material [15],[16]. Beside yeast strains, other chemical reactions occurring during fermentation can influence the final GSH content of the product, such as the incorporation in oxidation reactions with phenolic compounds as described by Sonni et al. [10],[11].

Although the addition of GSH as pure substance is allowed in must and wine, up to a maximum level of 20 mg L^−1^ according to the current OIV resolutions [17],[18], its usage can be costly. Moreover, a recent study by Wegmann-Herr and co-workers [19] has reported the formation of hydrogen sulfide (H_2_S) and other sulfur-related off-flavors favored by direct GSH addition.

On the other hand, the application of glutathione-enriched Inactive Dry Yeast preparations (GSH-IDY) seems to be promising in winemaking [20]. However, the action of these preparations and their influence on the sensory profile of wine are still unclear and further investigations are needed to optimize their formulation [21] as well as to elucidate the exact mechanism by which GSH-IDYs leads to increased GSH levels in wine [22].

For these reasons, the use of active yeast strains able to increase the GSH content in wine is still worthwhile. These strains are the direct producers of GSH *in situ*, i.e. in the fermentation tank. In such yeasts, GSH production must always be coupled with a robust fermentative aptitude and must be regulated to avoid any production of off-flavors. All the risks associated to inappropriate dosages of either pure GSH or IDY preparations are thus avoided. In addition, the direct production of GSH by yeasts results in an overall cheaper process, as it avoids or diminishes the requirement for exogenous GSH.

### 2.2. Glutathione in yeasts

In wild strains of the species *S. cerevisiae*, GSH represents more than 95% of the non-protein thiols with low molecular weight and can reach up to 1% of the cellular dry weight [23]. The biosynthesis of GSH is strictly connected to sulfate uptake in yeasts. In fact, sulfate is needed for cysteine biosynthesis, which is in turn one of the three components of GSH. The uptake of sulfates occurs through two specific membrane permeases, named Sul1p and Sul2p [24]. Then it is activated by adenylation yielding adenylyl sulphate (APS) and phosphorylated producing phosphoadenylyl sulphate (PAPS) [25]. PAPS is firstly reduced to sulfite and then to sulfide, which can be incorporated into the carbon chain of homocysteine. Subsequently, two transsulfuration reactions allow the interconversion of homocysteine to cysteine via the formation of the intermediate cystathionine [24]. GSH is synthesized in the cytosol by two enzymes γ-glutamylcysteine synthetase (encoded by *GSH1*) and GSH synthetase (encoded by *GSH2*) which act consecutively [26]. In the first reaction, γ-glutamylcysteine is synthesized by condensation of cysteine and glutamate; in the second reaction glycine is added for the synthesis of GSH [27].

Apart from the regulation of the cytoplasmic biosynthesis, GSH homeostasis is controlled by compartmentalization, degradation and consumption in different processes and import/export from the cell. GSH can be taken up from the extracellular environment through the Opt1p/Hgt1p transporter and secreted through the Gex1p GSH/proton antiporter. In fact, it was observed that the overexpression of the *GEX1* gene causes low cytoplasmic content of GSH due to the enhancement of glutathione excretion [28],[29].

The metabolism of GSH is also strictly connected to that of nitrogen. Mehdi and Penninckx [30] showed that nitrogen starvation triggers the expression of γ-glutamyltranspeptidase, a vacuolar enzyme that hydrolyses GSH to L-glutamate and cysteinylglycine.

Due to its low reduction potential, GSH also exerts a protective role against reactive oxygen species (ROS), either directly (i.e. non-enzymatically) reacting with the free radicals or by acting as a cofactor for redox enzymes such as glutathione peroxidase, glutathione reductase, glutaredoxin and glutathione S-transferase [31]. In this process GSH acts as an electron donor towards these species, undergoing an oxidative dimerization to glutathione (GSSG). The newly formed GSSG can then be brought back to the reduced form by glutathione reductase (GR) in the presence of NADPH [32]. The detoxifying activity of GSH is also exerted towards heavy metals such as cadmium, copper, zinc, silver, lead and xenobiotics, which are responsible for heavy oxidative stress on cells due to their ability to attract electrons [33].

## 3. Genetic Strategies to Improve Glutathione Production in Yeasts

Despite the high diversity in natural yeasts, winemakers are interested in novel strains with a combination of specific traits, which can confer a competitive advantage to the wine in terms of quality and consumer acceptance. Several attempts have been made to enhance GSH production by yeasts, although not all are suitable for winemaking.

In this paper some of the most common strategies successfully applied to obtain high-glutathione wine yeasts are reported.

### 3.1. Random mutagenesis

Random mutagenesis (RM) was one of the first techniques of genetic modification ever applied. The technique is based on the application of mutagens (chemical and physical ones) in order to enhance the natural mutation rate occurring in microorganisms. Once the mutations are produced, the phenotypes of interest are retrieved by applying either screening or selection procedures. The type and extension of the caused mutations are varied and depend on the applied agents, be they physical (e.g. UV, X-radiation, γ-radiation), chemical (e.g. alkylating agents, intercalating agents, base analogs) or biological (e.g. transposable genetic elements, viruses). Accordingly, mutations range from the minor modifications such as single base substitutions up to DNA frame-shifts and alterations of the chromosomal structure.

Random mutagenesis has a limited efficiency in wine yeasts, as they are usually diploid and homothallic [34],[35]. Useful phenotypic variants are produced at a slower rate compared to prokaryotes, as the recessive mutations are potentially masked by the dominant alleles on the homologous chromosomes, while sporulation followed by autodiploidization contributes to the purging of novel variants. However, homothallism also offers the opportunity to circumvent this issue: chemical mutagens, like ethyl methanesulphonate (EMS) and nitrous acid, as well as UV rays, can be directly applied to spores rather than to diploid cells. The mutation is produced on the haploid spores and subsequently brought to homozygosis by the auto-diploidization. The phenotypic effect will be displayed as a consequence, without any masking effect by the homologous allele [2],[36]. Wine yeasts were historically submitted to RM, in order to improve the flavor profile of wine. For instance, Rous et al. [37] and Giudici and Zinnato [38] used RM to reduce higher alcohol production by *S. cerevisiae* wine strains.

Several RM strategies have also been applied to isolate GSH over-producing mutants of *S. cerevisiae* and other species [39]–[42]. However, the exact mechanisms underlying higher GSH accumulation in those mutants was unclear. Recently, Nisamedtinov and co-workers [43] have obtained an UV-mutagenized *S. cerevisiae* strain able to produce a GSH concentration (i.e. 40–50 µmol g^−1^), several-folds higher than the one of the wild type strain. The authors proved that the higher accumulation of GSH in the mutant was caused primarily by the higher transcription rate of genes *CYS3* and *GSH1*, resulting in an increased biosynthesis of cysteine and a greater activity of the reaction step involving Gsh1p, respectively. Anyway, they also concluded that the exact mechanisms causing higher *CYS3* and *GSH1* transcription rate in the mutant remain to be elucidated.

### 3.2. Hybridization

Yeasts, especially those belonging to *Saccharomyces* spp. and, more in general, to the winemaking species, feature the major evolutionary and technical advantages of sexual recombination. The sexual hybridization techniques, both intra- and interspecific ones, are the most efficient way to generate artificial diversity in yeasts. In fact, even the overall genetic recombination and diversity of higher eukaryotes strictly rely on the meiotic division associated to the production of gametes. Meiosis shuffles the genome and produces alternative arrangements of genes, which are in turn reflected in alternative phenotypic outputs [44]. This process has deep implications for the development and improvement of industrially relevant traits in the winemaking field, such as fermentative performance, cryotolerance, thermotolerance and flavor profile [45]–[48].

Yeasts exposed to nutritional deficiency [49] are triggered towards sporulation, which entails the passage from a single diploid mother cell (2n) to four haploid daughter cells (n). After germination, thus-obtained spores can be induced to mate by attaching them to one another through micromanipulation.

**Figure 1.**
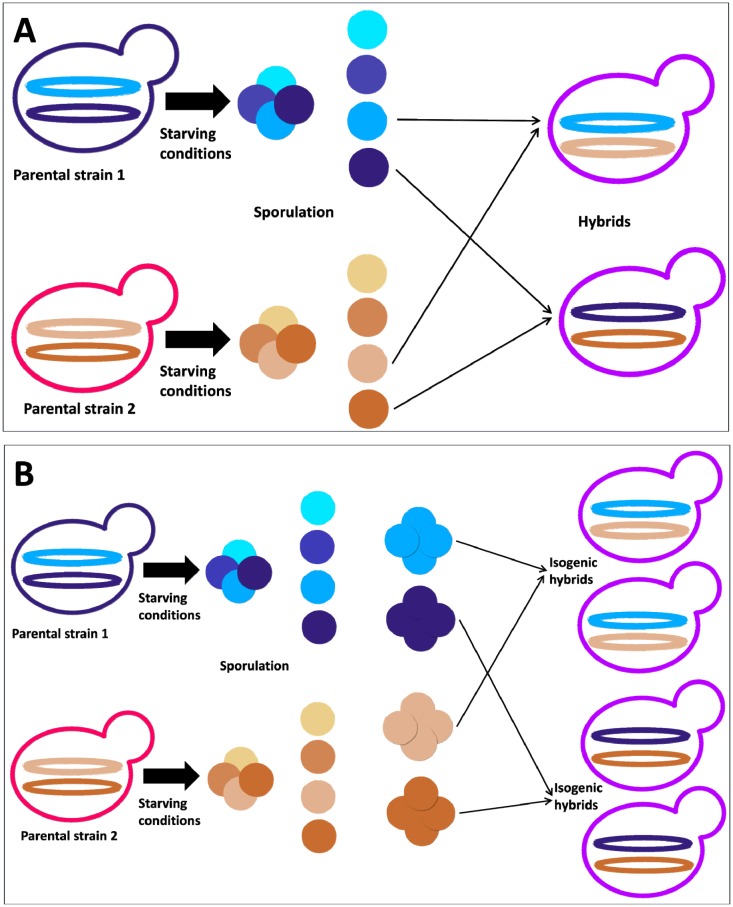
Breeding strategies for intra- and interspecies hybrids, based on spore-to-spore mating. (A) Hybrids obtained by direct mating. (B) Isogenic hybrids obtained using monosporic clones.

The spores obtained from a wild strain can either be directly used in a mating attempt or, alternatively, be cultivated as homozygous and diploid (for homothallic yeasts) or hemizygous and haploid (for heterothallic yeasts) strains. In the first case, the technique is termed “direct mating” and entails the highest degree of randomness and variability, given the putative high degree of heterozygosis in the wild genome (Figure 1A). In the second case, also termed “mating of monosporic clones”, the procedure starts from the constitution of homozygous lines from the wild progenitor (Figure 1B). Then, mating is performed among the different obtained lines. Such a strategy allows a more thorough exploration of the phenotypic space, although suffering from an overall lower time-efficiency [50] compared to “direct mating”, which is thus the most suitable for the rapid improvement of traits associated to quantitative trait loci (QTLs). In any case, genetic improvement programs based on monosporic cultures should comprise the extensive screening of desired phenotypic and genotypic strains in the progenitors [51].

Similar to fermentative vigor, ethanol yield and growth temperature optima, GSH production is controlled by QTLs. As the meiotic crossing-over associated to sporulation produces novel combinations at the level of the whole genome, attempts have been performed to improve the production of GSH via selective breeding programs.

In a recent study, Bonciani et al. [48] applied a direct mating approach involving many wine strains of *S. cerevisiae*. Although the overall procedure was aimed at the obtainment of robust winemaking strains, some important considerations concerning GSH production were incidentally highlighted. In fact, the work showed that, generally speaking, GSH production seems to be subjected to the dominant effect of one of the parents or on the additive contribution of genes. In the first case the produced titers were similar to those produced by one of the parents. In the second case the produced levels of GSH were intermediate between those of the parental strain.

Remarkably, also a case of hybrid vigor was recorded, with both parental strains greatly outdone in GSH production by their hybrid progeny. More specifically, the hybrid produced 137% more GSH compared to the best parent and 46% more GSH compared to the sum of the amounts produced by both parents, thus highlighting a synergic interaction among the involved genes. More in general, the article shows the huge advantages of direct mating when dealing with QTLs, with benefits in terms of enhanced genetic recombination and in terms of time-efficiency of the development process, as it managed to improve the target trait in a single generation.

### 3.3. Metabolic engineering

**Figure 2.**
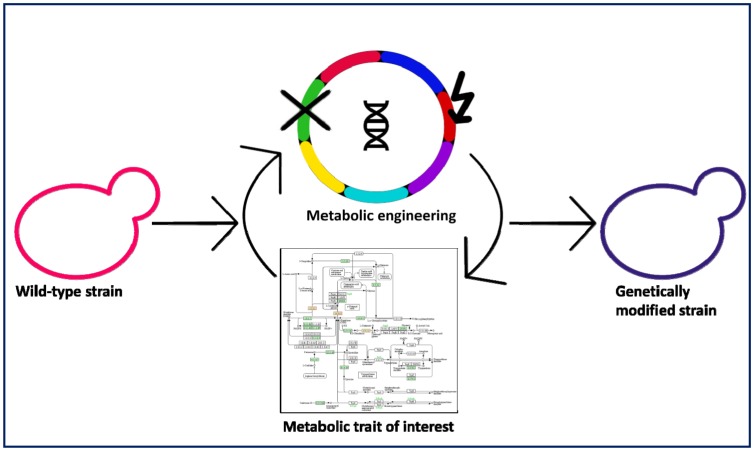
Schematic overview of metabolic engineering strategies for genetic improvement of yeast strains.

The term “metabolic engineering” (ME) addresses the rational and targeted modification of both genetic and regulatory mechanisms in a given microorganism to optimize the production of specific metabolites or the expression of industrially relevant traits (Figure 2).

It is a highly multidisciplinary approach, which was developed over the last decades and which strongly involves recombinant DNA techniques. The “omics” technologies (genomics, transcriptomics, proteomics and metabolomics) have given us the ability to perform fine genetic modifications. These techniques also provide us with a wealth of biological data, which currently drive our rational and knowledge-based approach to genetic modifications [52].

Due to the targeted nature of the modifications, ME strictly depends on the *a priori* knowledge of the molecular and regulatory network underlying either the considered traits or produced metabolites. Therefore, ME is easier to apply to model microorganisms, such as *S. cerevisiae* and *Escherichia coli* or, more in general, to all those microorganisms with a greater amount of produced literature.

*S. cerevisiae* is the most used yeast species in wine fermentations; being the model organism for eukaryotes, the species was the main subject of “omics” studies over the past century. Its genome was the first to be fully sequenced among the eukaryotes [53], which allowed the birth of several online databases over the years, encompassing genomic, proteomic and metabolomic data.

However, this technique suffers some major drawbacks for its employment in the oenological industry and more in general in food industry. The first is related to the nature of oenologically relevant traits, which are nearly all determined by the concerted expression of QTLs spread throughout the genome, thus requiring the application of recursive strategies of targeted genetic modification. For instance, the trait of ethanol tolerance seems to be determined by as many as 250 QTLs spread in the genome [1]. Secondly, we should not neglect the high degree of genetic redundancy and pleiotropy inherent to the eukaryotic organisms: in such a context, strictly deterministic predictions are hardly feasible, even in the model *S. cerevisiae*. Finally, predictions become even more difficult when shifting from the well-treaded field of *S. cerevisiae* to other less conventional wine yeasts.

The regulations concerning the microorganisms in food industry constitute a further drawback for the usage of ME, as it falls into the field of genetic modification techniques. However, this is a minor problem, as the usage of GMOs applied to the winemaking industry is negatively perceived by the customers. For instance, a survey conducted in the European Union showed that 95% of the population would not choose to knowingly buy a GMO-derived product [54]. Nevertheless, in other contexts, such as in the U.S.A., the application of genetically modified yeasts to the winemaking field is allowed [55].

Although none of them within the winemaking field, many attempts have been performed to apply ME to GSH production in yeasts. The simplest examples encompass the simple transformation of expression vectors containing glutathione reductase [56].

In other cases, the approach was based on a more thorough evaluation of the metabolic networks involved in GSH production. In particular, the development of genetically engineered strains overexpressing either GSH biosynthetic enzymes or the key enzymes in sulfur assimilation pathways, aimed at increasing cysteine biosynthesis, were reported in literature [57],[58].

Moreover, some authors reported ME strategies for improving GSH production in *S. cerevisiae* strains, required to survive in stressful environments like those occurring in the simultaneous saccharification and fermentation of lignocellulosic feedstocks [59],[60]. They showed that the enhanced GSH content in *S. cerevisiae* strains had a relevant influence on their robustness.

### 3.4. Evolutionary engineering

The improvement of wine yeasts by evolutionary engineering, also referred to as “adaptive laboratory evolution” or “directed laboratory evolution”, is a widely-used approach. Evolutionary engineering is based on miming selection mechanisms active in nature. This is accomplished through the controlled application of a selective pressure on the microbial population, in order to select evolved strains with specific phenotypes [61],[62]. The strength of the strategy is that no prior genetic knowledge is required to obtain new evolved strains.

**Figure 3.**
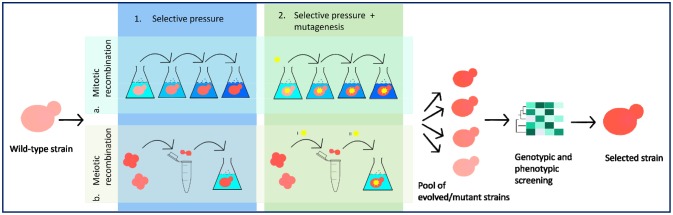
Adaptive evolution strategies from the wild type strain to the selected evolved strain. 1a. Mitotic recombination under selective pressure, with increasing selective pressure; 2a. Mitotic recombination with initial mutagenesis followed by selective pressure conditions; 1b. Meiotic recombination applying selective pressure, starting from spores, followed by spore conjugation and application of selective pressure; 2b. Meiotic recombination under selective pressure with mutagenesis, which can be applied in phase I (on spores) or phase II (on newly-generated zygotes).

Generally, the evolutionary engineering strategies consist of two basic steps: the strain randomization by mutation and/or recombination, and the selection of evolved strains [63]. These strategies allow genetic drifts of the whole population, caused by mitotic or meiotic recombination events and/or accumulation of natural or induced mutations. The mentioned mutations/recombinations are selected during cell growth, which favors the advantageous ones (Figure 3).

Although the basic laboratory procedure for adaptive evolution has been performed in a similar way for many years, new experimental approaches have been recently developed to improve the successful retrieval of the evolved strains and for providing a more rapid screening of those of interest [64].

Cell growth under desired conditions can be performed in batch culture or in continuous culture systems such as chemostats or turbidostats [44]. In the latter case, it is possible to set defined technological parameters alongside the specific selective pressure, in order to select evolved strains, which possess both the specific phenotype and a good fermentative performance, sometimes even better than the parental one.

Evolutionary engineering is particularly valuable to improve strains used in food and beverage technologies, where the application of genetically modified organisms (GMO) is prohibited or limited by legal restrictions. Therefore, since they have not undergone artificial genomic modifications, the use of yeast strains obtained by adaptive evolution approaches has a high degree of acceptance by the consumers, even in winemaking.

Several examples of yeasts with improved oenological properties, such as tolerance to high ethanol concentrations, low sulfite and sulfide production, resistance to various stresses or efficient substrate utilization have been described in literature [63],[65]–[69]. However, the application of evolutionary engineering might also lead to trade-off phenomena, consisting in the acquisition of the desired trait at the expenses of another [70].

A crucial step in evolutionary engineering strategies is the screening of evolved strains. They are cultured under a defined combination of conditions in order to select those specifically adapted and possessing the desired properties. Indeed, the screening of the evolved strains can be easily performed for improved phenotypes which are directly selectable, such as the ones linked to growth, e.g. stress resistance and temperature growth. Otherwise, the screening process turns out to be time-consuming, because a large number of strains need to be screened individually to retrieve the evolved ones expressing the desired phenotype, as it is not directly selectable.

To overcome this drawback, a possible strategy is to find a selectable phenotype that indirectly allows a rapid screening of the desired evolved strains. De Vero and co-workers [63] reported evidence for the effectiveness of this approach. In particular, they exploited the resistance to chromate Cr(VI) and molybdate Mo(VI), both sulfate analogues, as selectable phenotype to rapidly select *S. cerevisiae* strains impaired in the sulfate assimilation pathway and characterized by a low production of SO_2_ and H_2_S.

Moreover, their designed evolution-based strategy was recently applied to generate evolved *S. cerevisiae* strains with enhanced GSH production by activation of the yeast common metal response that involves GSH biosynthesis [69]. According to the strategy, at first random spore hybridization of the strain UMCC 855 (=21T2) was achieved and then a high toxic concentration of Mo(VI) (up to 5 mM) was applied as specific selective pressure. Molybdate, as well as other toxic oxyanions like Cr(VI) and Se(VI), is structurally similar to sulfate. Furthermore, it can enter the yeast cell through Sul1p and Sul2p high-affinity sulfate permeases. Therefore, a mutation in these permeases can confer resistance to the cell [71],[72],[73]. Although this is one of the most important mechanisms of resistance, even others could be involved. In particular, the biosynthesis of GSH, which is strictly connected to the sulfate assimilation pathway, has an essential role in the cellular defense against oxidative stress and metal toxicity [31],[73].

**Figure 4.**
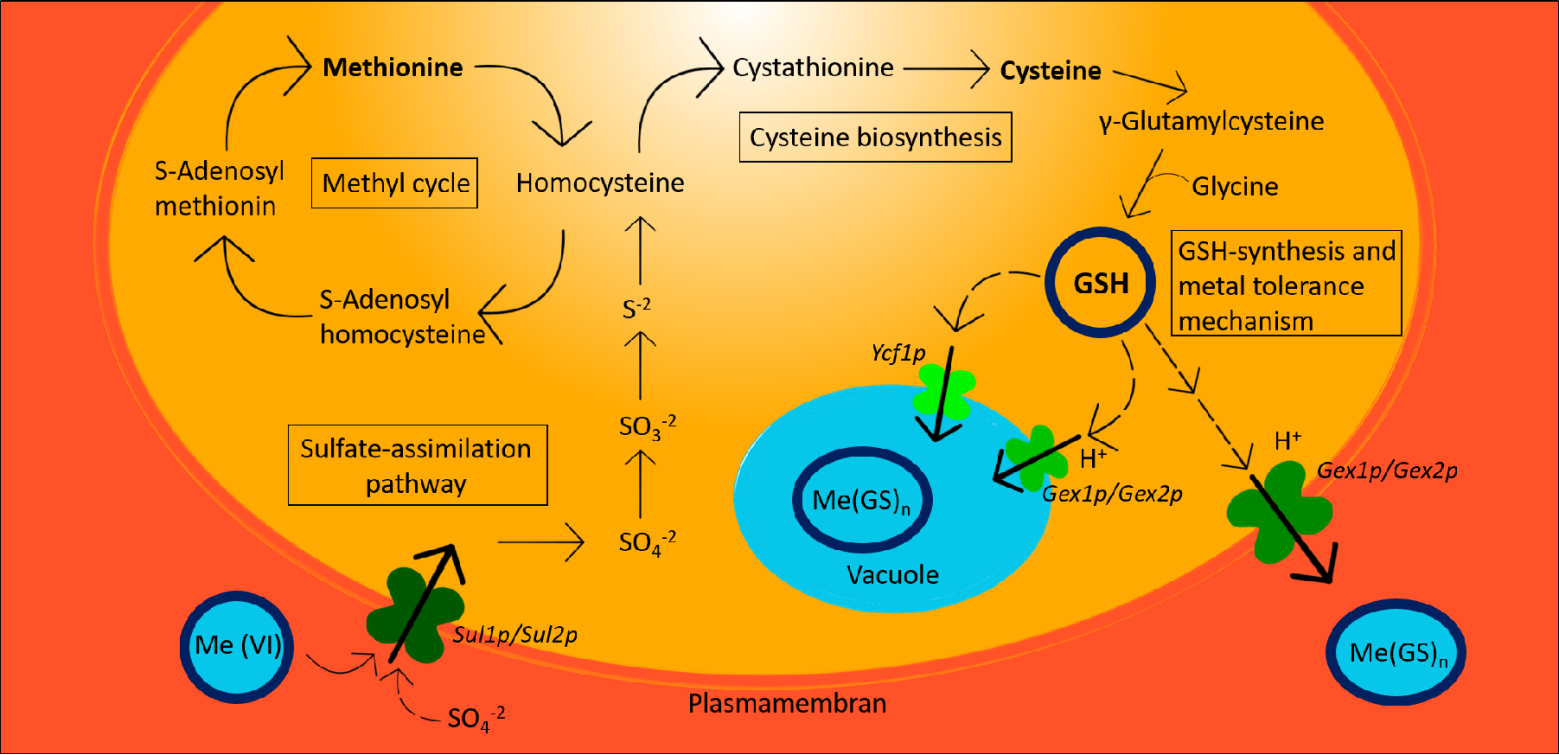
Scheme representing the metabolism of sulfur, the synthesis of glutathione and the GSH-mediated metal tolerance mechanism in *S. cerevisiae*. Me(VI): toxic metal oxyanion, sulfate analogue; Sul1p/Sul2p: sulfate transporters; Gex1p/Gex2p: yeast glutathione exchangers; Ycf1p: vacuolar glutathione S-conjugates pump; Me(GS)n: Metal-GSH complex.

In figure 4a schematic model for GSH biosynthesis in *S. cerevisiae* is reported. The process involves two ATP-dependent steps: firstly, cysteine is linked with glutamate by γ-glutamylcysteine synthetase (encoded by *GSH1*) to form γ-glutamylcysteine. Secondly, glycine is added to this intermediate product by glutathione synthetase (encoded by *GSH2*) to form the final product [42],[74]. GSH can chelate heavy metals by exploiting the cytosolic glutathione S-transferase enzyme, which enables the formation of a metal-GSH complex (Me(GSH)n). The Me(GSH)n complex is recognized as substrate by specific transporters (Ycf1p and Gex1p) leading to either vacuolar sequestration or export outside the cell [25],[75],[76].

Once inside the vacuole, the complex can be decomposed by the enzyme Υ-GT, the same enzyme involved also in GSH degradation and, thereafter, by other peptidases, restoring the amino acids in the cytoplasm. Mentioned aminoacids can then be used for the *de novo* synthesis of GSH [77].

By applying this strategy to the yeast strain UMCC 855, a pool of eight different evolved strains resistant to Mo(VI) were obtained, among which the strain UMCC 2851(=Mo21T2-5) resulted capable of enhancing GSH content in wine, with an increase of 100%, compared to the parental strain [69].

An adaptive evolution approach similar to those previously described, although not specific for the winemaking field, has been recently applied by Patzschke and co-workers [78], which used acrolein, a reactive α,β-unsaturated aldehyde highly toxic for cells, as a selection agent in order to obtain strains with an enhanced glutathione accumulation phenotype, coupled with an acrolein resistance phenotype. Moreover, other authors reported the resistance to highly toxic compounds such as ethionine, 1,2,4-triazole and sodium cyanide as a screening method for selection GSH-overproducing strains [42].

Strains obtained by evolutionary engineering can be further investigated in order to understand the relation between genotype and phenotype. Indeed, the comprehension of the genetic determinants and the molecular mechanisms underlying the desired feature can be used for further selection or breeding programs, combining evolutionary approaches with metabolic engineering programs.

This combined strategy, called “inverse metabolic engineering” has been receiving an ever-growing interest in the last years. It consists in a “bottom-up” approach that achieves both the selection of useful evolved strains and the assessment of the genetic changes conferring the phenotype of interest. The three components of this approach are: (i) the selection of the evolved strains through evolutionary engineering strategies; (ii) the study of the molecular basis for the trait of interest by using genetic mapping or “omics” techniques; (iii) the application of the gained knowledge to metabolically engineer the target strain [79],[80],[81].

## 4. Conclusions

Winemaking is a complex process, in which many factors, including grape variety and must quality, technological procedures, alcoholic fermentation processes and involved microorganisms contribute to obtain the final product [35],[82]. Despite the advances of metabolic and cell engineering strategies, the application of these strategies in wine genomics is still rarely considered.

Natural evolutionary processes in wine have gradually allowed the yeast physiology and genome to cope with the harsh and cyclically changing conditions of fermentation and with the long periods that separate successive vintages. This is mainly due to their propensity for genetic/genomic alterations, allowing their properties to change and adapt to fluctuating environmental conditions. On the other hand, laboratory and industrial wine yeasts have also many distinctive features that allow them to adapt to industrial conditions, where multiple stresses occur during fermentation, such as low pH, high osmolarity, high SO_2_ content, nutrient limitation, temperature variations and ethanol toxicity [83],[84]. Because of this wide range of stressors and the different competing strains involved in most industrial environments, it is no longer sufficient to simply improve the yeast phenotype for a single condition [64].

A challenge for evolutionary engineers is to achieve continuous improvements in tools and methodologies requested for the rapid constitution of yeasts with these multiple complex phenotypes. These yeasts should represent the next generation of oenological industrial starters, designed to address specific market sectors and complying with the requirements of both wine consumers and producers. The new strains should possess both, a basic fermentative robustness and the added value provided by more specific traits of interest. In this regard, the application of special wine yeasts, improved for GSH production, will provide the benefits in terms of product stability and thus shelf-life, still maintaining a predictable and quick fermentation process.
